# Pseudomyxoma Peritonei in a Case of Carcinoma Cervix: Subtle Finding With Implications on Management and Prognosis

**DOI:** 10.1155/2024/3066063

**Published:** 2024-08-01

**Authors:** Sarita Kumari, Suvidya Singh

**Affiliations:** Department of Gynaecologic Oncology National Cancer Institute All India Institute of Medical Sciences 110029, New Delhi, India

**Keywords:** adenocarcinoma, carcinoma cervix, pseudomyxoma peritonei

## Abstract

Pseudomyxoma peritonei (PMP) is a well-known entity in gastrointestinal and ovarian tumors of mucinous histology. It has important implications on prognosis depending on whether seen in conjunction with a benign or a malignant tumor. In the current report, we describe a case of PMP in a case of advanced endocervical adenocarcinoma of the cervix which was managed surgically.

## 1. Introduction

Pseudomyxoma peritonei (PMP) is a clinical condition characterized by progressive intraperitoneal accumulation of mucinous ascites. The diffuse ascites contains gelatinous material along with implants on peritoneal surfaces. The presence of mucin is a prerequisite for clinical diagnosis [1]. It was first reported in 1842 and was believed to arise from an ovary [2]. Subsequent reports also confirmed an ovarian origin, and it was identified as a “gelatinous disease of the peritoneum” [2]. However, in 1901, it was first described as a ruptured appendicular cyst [3]. The reported incidence is 2/10,000 laparotomies with a female preponderance usually in their 50s [3]. The most common etiology is a mucinous tumor of the appendix, particularly mucocoele [1]. Less commonly, it has been seen in mucinous tumors of the gall bladder, colon, rectum, stomach, pancreas, and urachus. When seen in association with ovarian tumors, it is still debatable whether the primary is ovary or appendix with ovarian metastasis [4]. Regardless of the origin, histology is always a mucinous tumor. However, PMP occurring in the case of endocervical adenocarcinoma has not been reported so far. We describe here a case of peritoneal dissemination of mucin in a patient diagnosed with endocervical adenocarcinoma.

## 2. Case

A 44-year-old female presented with a chief complaint of intermenstrual bleeding for the last 1 year. Her prior cycles were regular. There was no complaint of discharge per vaginum or pelvic pain. Her prior medical history was unremarkable. On clinical examination, there was an ulceroproliferative growth involving the anterior and posterior lip of the cervix of size measuring ~7.0 cm, extending up to the lateral pelvic walls along with palpable cystic masses in the bilateral adnexa. A cervical punch biopsy was suggestive of endocervical adenocarcinoma, immunopositive for p16. Contrast-enhanced computed tomography (CECT) scan done for her was suggestive of a 6.0 cm lesion in the cervix with ill-defined fat planes with bladder and rectum; solid-cystic lesion in left adnexa of 6.8 × 4.4 cm and another cystic lesion of 4.4 × 3.0 cm in right adnexa. There were multiple enlarged iliac and paraaortic lymph nodes measuring 1.3 and 1.2 cm, respectively, and omental nodularity. Radiologically, there was ascites as well. Serum CA125 and carcinoembryonic antigen (CEA) were raised to 308 U/ml and 24.6 ng/ml, respectively, and CA19.9 was within the normal range.

**Figure 1:**
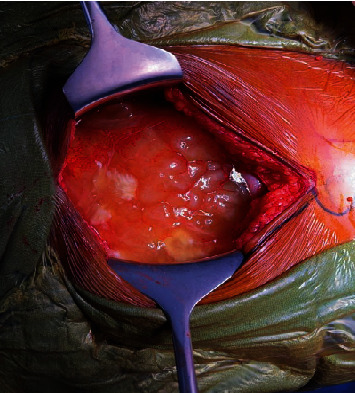
Mucinous ascites filling the peritoneal cavity.

**Figure 2:**
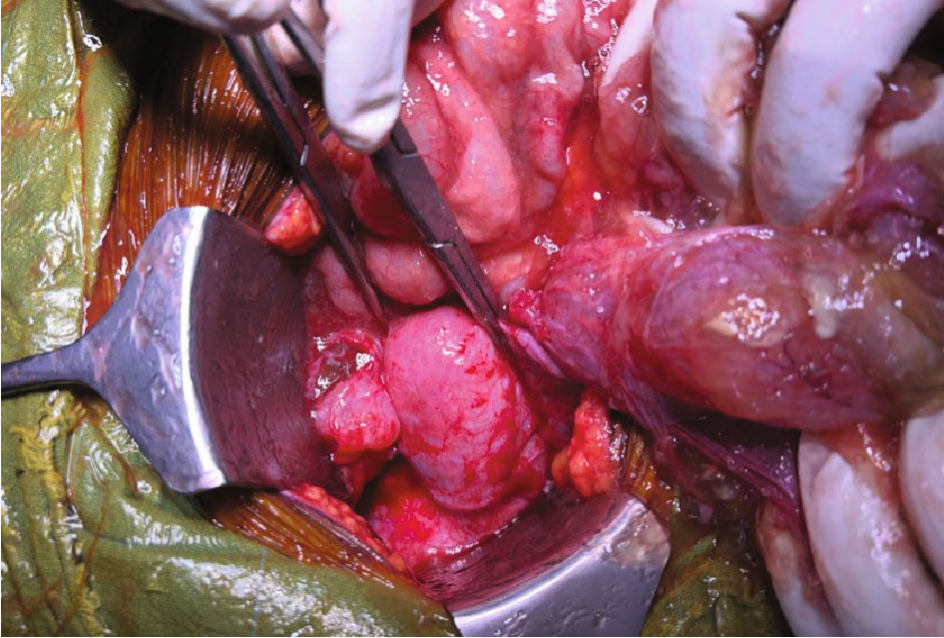
10 × 10 cm cyst with gelatinous mucin in left ovary.

At this point, there was a diagnostic dilemma regarding the patient either having dual primaries (carcinoma cervix stage IIIC2 along with ovarian carcinoma) or carcinoma cervix with metastasis to ovaries. In order to reach a correct diagnosis, we went ahead with an ovarian mass biopsy. We performed an abdominal ultrasound-guided biopsy from the ovary. The biopsy was reported as mucinous carcinoma with cells immunopositive for CK7 and p16 and negative for CK20, suggesting likely metastasis from endocervical adenocarcinoma. After a detailed discussion in the tumor board, the patient underwent 8 cycles of 3 weekly carboplatin and paclitaxel. Postchemotherapy CECT was suggestive of partial response; mass lesion in the cervix, omental deposits, and left adnexal lesion had resolved. Right adnexal cystic lesion of 7.0 cm persisted along with moderate ascites. Postchemotherapy CA125 decreased to 10 U/ml. In view of a good response to chemotherapy and a persistent adnexal mass, we planned her for laparotomy. Intraoperatively, we noted mucinous ascites filling the peritoneum (Figure 1); the left ovary was replaced by a 10 × 10 cm cyst filled with gelatinous mucin (Figure 2), and the right ovary also had a 5 cm cyst filled with mucin. The omentum and bowel loops were completely covered with mucin. We performed a salvage hysterectomy with bilateral salpingo-oophorectomy, omentectomy, and appendicectomy. Thorough peritoneal lavage was done with 5% dextrose.

**Figure 3:**
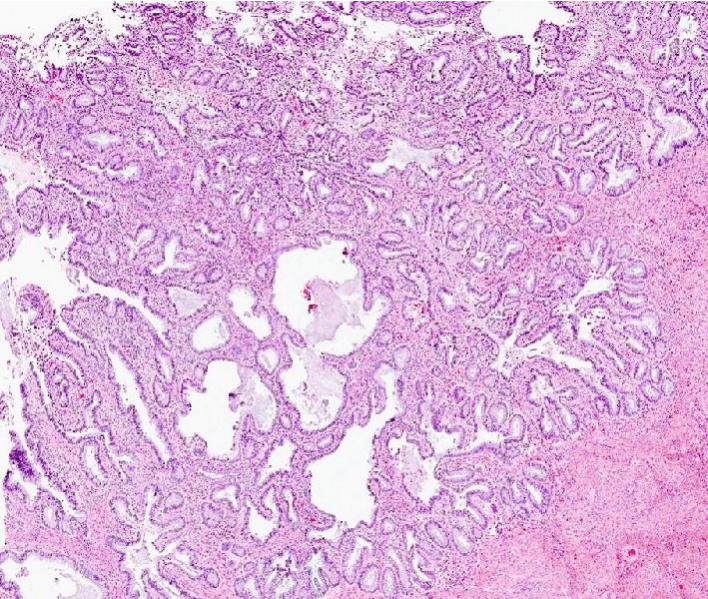
Histopathology image of cervix showing adenocarcinoma at 10x magnification.

**Figure 4:**
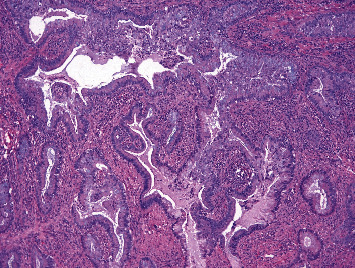
Histopathology image of ovary cervix showing adenocarcinoma with mucin at 40x magnification.

Histopathological evaluation of the specimens suggested endocervical adenocarcinoma, silva pattern B, involving the lower segment of the uterus and 50% of the myometrium (Figure 3). The tumor cells were immunopositive for p16. Lymphovascular invasion and perineural invasion were not seen. Moderate amounts of tumor-infiltrating lymphocytes were noted. Sections from bilateral ovaries showed features of metastatic endocervical adenocarcinoma (Figure 4). Bilateral fallopian tubes, appendix, and lesser omentum were free of tumors. Greater omentum showed metastatic tumor deposit. Since the patient had already received chemotherapy, it was planned to keep her on follow-up and rechallenge chemotherapy or adjuvant radiotherapy in case of recurrence. Eight months postsurgery, she had a recurrence in pelvic nodes and received pelvic radiation. At present, she is alive and disease-free at 12 months postprimary treatment.

## 3. Discussion

**Figure 5:**
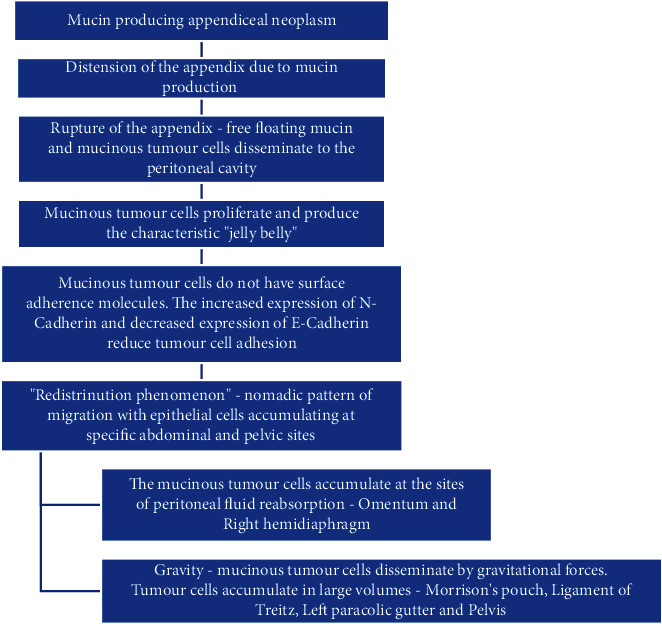
The pathophysiology of pseudomyxoma peritonei. *Source:* Kostov et al. [5].

PMP is characterized by the presence of mucinous ascites within the peritoneal cavity. Its origin, clinical presentation, and prognosis have always been a subject of debate. The pathophysiology of PMP is depicted in Figure 5 in the flow chart below [5]:

Most studies have identified PMP to be of an appendiceal origin (14/15 cases; 93%); however, there are case reports of its origin from low-grade/borderline or malignant mucinous neoplasm of the ovary, urachus, and other gastrointestinal tract organs [6–9]. The pathological classification of PMP is depicted in Table 1 [5].

In a recent literature review of 35 published cases of PMP, the mean age at presentation was 47.8 years. The presenting symptoms were abdominal pain, nausea, vomiting, fatigue, urinary abnormalities, and appendicitis. In the majority of cases, diagnosis was made on CECT or magnetic resonance imaging (MRI) followed by histology verification of the primary tumor [10]. Tumor marker CEA has been reported to be increased in 50% to 70% and CA19-9 in 55% to 65% of patients, respectively [11].

In the study by Nayanar et al., abdominal distension was the commonest presentation. Synchronous detection with primary tumor was seen in 60% of cases. Overall survival was 37% at 18 months among 15 cases in their study [6]. When the origin is appendiceal, large-scale studies have reported favorable survival outcomes with cytoreductive surgery. In a 10-year retrospective study from Japan, among 989 patients treated with a curative-intent cytoreductive surgery, complete cytoreduction was achieved in 702 patients (71%) with a major complication rate of 17%. The median overall survival was 92.9 months [12].

It is rare for cervical cancer to be associated with psedomyxoma peritonei, and we could achieve only one prior report of such an analogy. In 2008, Gatalica, Foster, and Loggie reported a case of mucinous peritoneal metastasis 8 years after a hysterectomy in a 60-year-old patient who had cervical adenocarcinoma [13]. In their case, the authors were not able to identify any other primary site for the peritoneal metastasis. The peritoneal lesion tested positive for high-risk human papilloma virus both at episomal and integration levels which indicated abdominal and pelvic cancer contamination probably at the time of hysterectomy.

The cause of cervical adenocarcinoma disseminating to the omentum and leading to PMP is not very clearly understood, but one of the theories explaining it could be retrograde menstruation from tubes as occurs in serous ovarian tumors [14]. In a similar way, it may be possible that adenocarcinoma cells from the endocervix very rarely spread into peritoneal space through retrograde menstruation. In our case, the patient was 44 years old and menstruating with irregular cycles. Large amounts of mucinous fluid discharged into the uterus may be forced into the fallopian tubes and then expressed into the free peritoneal space. The fluid containing tumor cells soon contaminated the peritoneum leading to extensive peritoneal metastasis and ovarian involvement in our case.

Another theory explaining this condition could be uterine perforation resulting in direct inoculation of cancer cells into peritoneal space. However, in our case, the dissemination was diagnosed prior to surgical intervention and there was no prior history of any surgery. Complete cytoreduction along with hyperthermic intraperitoneal chemotherapy (HIPEC) is the standard of care for a PMP from malignant causes; at the time of the current case, the facility for performing HIPEC was not available at our center. Hence, we performed a thorough peritoneal lavage with 5% dextrose as it has been reported to prevent and delay recurrences of pseudomyxoma [15, 16]. This technique has also been performed in a large number of cases at our center in mucinous tumors with intraperitoneal spillage (unpublished data).

## 4. Conclusions

PMP in association with mucinous tumors of the gastrointestinal tract and ovary is a well-known entity. However, rarely, it could be seen in mucin-secreting adenocarcinoma of the cervix; however, the cause remains unknown at present. An incidental finding during laparotomy could pose dilemmas in diagnosis, management, and prognosis in an already advanced-stage tumor.

## Figures and Tables

**Table 1:** Pathological classification of pseudomyxoma peritonei.

**Diagnostic terminology**	**Histological characteristic**
Acellular PMP	Mucin without neoplastic epithelial cells, confined to the vicinity of the organ of origin or distant from it

LAMN PMP	Low-grade cytologic atypia Epithelial component scanty (< 20% tumor volume)
	Few mitoses
	Invasion into underlying organs is generally of the “pushing type”

HAMN PMP	High-grade cytologic atypia
	Infiltrative invasion into adjacent tissue
	Angiolymphatic or perineural invasion
	Cribriform growth
	Numerous mitoses

HAMN PMP with signet ring cells	Neoplasm with signet ring cell component (minimum 10% signet ring cells)

*Note: Source:* Kostov et al. [5].

Abbreviations: HAMN, high-grade appendiceal mucinous neoplasm; LAMN, low-grade appendiceal mucinous neoplasm; PMP, pseudomyxoma peritonei.

## Data Availability

The authors have nothing to report.

## Nomenclature

CA125: cancer antigen 125
CEA: carcinoembryonic antigen
CECT: contrast-enhanced computed tomography
CK7/20: cytokeratin
MRI: magnetic resonance imaging
